# Livestock Antibiotics Use and Antimicrobial Resistance

**DOI:** 10.3390/antibiotics14060621

**Published:** 2025-06-19

**Authors:** Elliot Enshaie, Sankalp Nigam, Shaan Patel, Vikrant Rai

**Affiliations:** 1 College of Osteopathic Medicine of the Pacific, Western University of Health Sciences, Pomona, CA 91766, USA; elliot.enshaie@westernu.edu (E.E.); sankalp.nigam@westernu.edu (S.N.); shaan.patel@westernu.edu (S.P.); 2 Department of Translational Research, Western University of Health Sciences, Pomona, CA 91766, USA

**Keywords:** antibiotics, antimicrobial resistance, bacterial strains, livestock, environment

## Abstract

**Background/Objectives**: Antibiotic resistance or antimicrobial resistance (AMR) in livestock is a growing global concern that threatens both human and animal health. The overuse and misuse of antibiotics in livestock production have led to an increased propensity for the development of AMR bacterial strains in animals, which can be spread to humans through the consumption of contaminated animal products, direct contact, or environmental exposure. This review aims to summarize the development and transmission of AMR in livestock, explore its underlying mechanisms and impact on human and animal health, and discuss current practices and potential strategies for mitigation and prevention. **Methods**: For this narrative review, we searched articles on PubMed and Google Scholar using the terms antibiotic resistance, livestock, and environment, alone or in combination. **Results**: The history of antibiotic use in livestock and its link to increased AMR, along with the involved mechanisms, including the enzymatic breakdown of antibiotics, alterations in bacterial targets, horizontal gene transfer, and efflux pumps, are important. Antibiotics in livestock are used for growth promotion, disease prevention and control, and metaphylactic use. The role of livestock and the environment as reservoirs for resistant pathogens, their impact on human health, chronic infections, allergic reactions, toxicity, and the development of untreatable diseases is important to understand AMR. **Conclusions**: Given the widespread use of antibiotics and the potential consequences of AMR, collaborative global efforts, increased public awareness, coordinated regulations, and advancements in biological technology are required to mitigate the threat AMR poses to human and animal health. Regulatory solutions and the development of new therapeutic alternatives like antimicrobial peptides and bacteriophage therapy, and preventive measures such as DNA and mRNA vaccines, are future perspectives.

## 1. Introduction

Antibiotics have played a crucial role in modern medicine and are described as one of the most successful forms of therapeutics in the history of medicine [1]. The development of anti-infective drugs is commonly credited to Paul Ehrlich, who developed the arsenic-based drug, Salvarsan, to treat syphilis [2]. This was followed by the discoveries of sulfonamides, penicillin, and tyrocidine, which helped initiate the Golden Age of antibiotic discovery from the 1940s to mid-1960s [3]. This period transformed medicine by enabling safer medical treatment and surgeries, reducing deaths from infectious diseases, and improving overall public health. However, the overuse and misuse of antibiotics set the stage for antimicrobial resistance (AMR) [4].

The agricultural use of antibiotics began with synthetic sulfonamides, with the drug Prontosil [5]. This began the practice of adapting human antibiotics for livestock, most notably the use of penicillin to protect cows and milk production during World War II. This fostered connections between pharmaceutical and feedstuff companies for the mass medication of entire herds and flocks to curb the disease in these populations [6]. As antibiotic use proved to be successful, non-therapeutic antibiotic use emerged as a lucrative practice. These therapies included increasing animals’ weight gains to satisfy the increasing demand for meat [7,8]. New antibiotic applications crossed the Atlantic and became pervasive throughout Europe in the 1950s to combat various bacterial diseases and function as preservatives [9]. The overuse of antibiotics was readily apparent, which started concerns for AMR as early as the mid-1950s [10].

The issue of misuse of antibiotics and AMR in livestock is more prevalent in developing countries due to unregulated antibiotic sales, poor healthcare infrastructure, the overuse of antibiotics for non-therapeutic growth promotion, indiscriminate and irrational use without following the withdrawal period, the contamination of animal feed with the excreta of treated animals, extra-label dosages for animals, poor infection control, environmental contamination, and the use of unlicensed antibiotics [11,12,13].

Today, many antibiotics are similarly administered to both individual animals and entire herds/flocks to treat bacterial infections such as respiratory diseases or enteric infections. Antibiotics are often administered prophylactically to healthy animals for disease prevention in crowded environments [14]. They are also used in metaphylactic use, which refers to administering antibiotics to an entire group of animals when a few individuals exhibit signs of infection to contain the disease spread [15]. Over half of all antibiotic use is for animal husbandry, mainly for non-therapeutic use, such as growth promotion [16]. Additionally, these antibiotics are consistently detected in low levels in the gastrointestinal tracts of livestock [17]. This exerts a selective pressure on bacteria to acquire antibiotic-resistant genes and increases the abundance of these genes in gastrointestinal bacterial populations [18]. The excretion of these bacteria leads to local contamination of the soil and water supply and facilitates the spread to humans. Environmental contamination from livestock antibiotics is one of a few key pathways, along with the use of antibiotics in healthcare facilities, contributing to AMR, a critical public health concern [19,20].

Antimicrobial resistance (AMR) represents a growing and interconnected threat to both human and animal health. The inappropriate and prolonged use of antibiotics, particularly in the management of chronic conditions, has been a major driver of resistance development across both medical and veterinary domains. This convergence accelerates the emergence and spread of resistant pathogens, diminishing the efficacy of existing antimicrobial agents. Consequently, common infections may become increasingly difficult or impossible to treat, routine surgical procedures may carry heightened risk, and therapeutic options may become severely limited. In recognition of the magnitude of this challenge, the World Health Organization (WHO) has designated AMR as a global health emergency and identified it among the top ten threats to global health, with the potential to cause millions of deaths annually [21].

In this comprehensive review, we have discussed the development and transmission of AMR in livestock, followed by its underlying mechanisms and impact on human and animal health. The review also discusses the current global practices and potential strategies for mitigation and prevention.

## 2. Methods 

For this narrative review, we searched articles only in the English language on PubMed and Google Scholar using the terms antibiotic resistance, livestock, and environment, alone or in combination. After selecting the related articles, only abstracts, duplicate articles, and short summaries were removed. Finally, 108 articles were selected to include in this review.

## 3. Antibiotic Use in Livestock

Approximately 50% of antibiotics produced globally are used for livestock [22]. In 2020, the top five consumers of veterinary antimicrobials were China, Brazil, India, the USA, and Australia, which comprised 58% of global antimicrobial use (AMU) [23]. Tetracyclines are the most commonly used antimicrobial and are predicted to increase by 9% by 2030. Based on current trends, the overall global AMU is predicted to increase by 8.0% by 2030 [23]. The World Health Organization (WHO) classifies antibiotics that both humans and animals use into categories based on their importance to human medicine: Highest Priority Critically Important Antibiotics (HPCIAs), Critically Important Antibiotics (CIAs), Highly Important Antibiotics (HIAs), and Important Antibiotics (IAs) (https://www.avma.org/news/; accessed on 29 April 2025). Higher-priority antibiotics are essential for treating serious human infections that often have few or no alternative treatments. Thus, resistance to these antibiotics poses a serious threat to public health.

Macrolides are a CIA class of antibiotics that are considered critically important as a treatment for severe cases of campylobacteriosis in humans. Macrolides inhibit bacterial protein synthesis by binding to the bacterial 50S ribosomal subunit and preventing peptidyltransferase from adding amino acids to the growing peptide chain. Common macrolides for livestock include Tylosin, Tulathromycin, Tilmicosin, and Erythromycin. In livestock, they maintain a critical role in the treatment and control of respiratory infections and liver abscesses [24]. In the USA, the majority of macrolide use in livestock in 2017 was for cattle (59%) and pigs (40%). Macrolides are used in swine for the treatment and prevention of *Lawsonia intracellularis* infection and for the treatment and control of bovine respiratory disease complex caused by *Mannheimia haemolytica*, *Pasteurella multocida*, *Mycoplasma bovis*, and *Histophilus somni* in cattle [25]. 

Fluoroquinolones (FQs) are a broad-spectrum, HPCIA class of antibiotics that include ciprofloxacin, levofloxacin, and moxifloxacin, among others. In the USA, danofloxacin and enrofloxacin are the two Food and Drug Administration (FDA)-approved FQs for use in food-producing animals, and their uses include treating and controlling respiratory disease in cattle and swine. FQs elicit their antimicrobial effect by converting gyrase and topoisomerase IV into toxic enzymes that fragment the bacterial chromosome.

Tetracyclines are one of the most widely used classes of antibiotics in cattle, pigs, sheep, and poultry. They possess a broad spectrum of molecular activity and offer a relatively low cost. Tetracyclines exert their bacteriostatic effect by inhibiting the 30S ribosomal subunit and inhibiting cell growth and proliferation. Although there are more than 20 types of available tetracyclines, the commonly used ones include tetracycline, chlortetracycline, oxytetracycline, and doxycycline [26,27]. In cattle and calf feeding trials with chlortetracycline, the animals showed increased daily weight gain, a significant reduction in the incidence of liver abscesses, and increased carcass value [28]. In swine, cattle, and calves, tetracyclines can be used to increase average weight gain and feed efficiency, abate abscesses, and treat bacterial enteritis by Escherichia coli and pneumonia caused by *P. multocida* [29]. However, the misuse and overuse of antibiotics, particularly long-term sub-therapeutic use, may place selective pressure on antibiotic resistance in livestock populations. In the USA, tetracycline use for growth promotion has not been allowed since 1 January 2017. Their use is restricted to therapeutic use only, and they have been active against *Mycoplasma*, *Chlamydia*, *Pasteurella*, *Clostridium*, *Ornithobacterium rhinotracheale*, and some protozoa [29]. 

Antibiotic resistance can arise due to spontaneous mutation and is conferred by three fundamental mechanisms: antibiotic deactivation, extrusion through efflux pumps, and protection of targets like ribosomes. Antibiotic-resistant genes provide resistance to nine major classes of antibiotics: tetracyclines (tet), sulfonamides (sul), β-lactams (bla), macrolide-lincosamid-streptogramin B (erm), aminoglycosides (aac), FCA (fluoroquinolone, quinolone, florfenicol, chloramphenicol, and amphenicol) (fca), colistin (mcr), vancomycin (van), and multidrug (mdr). The most frequently detected classes in livestock waste include tet, sul, erm, fca, and bla [16,30].

## 4. Antibiotic Resistance in Livestock

As discussed previously, antimicrobial resistance (AMR) occurs when bacteria face selective pressure to acquire antibiotic-resistant genes to survive the effects of antibiotics. AMR mechanisms are generally categorized into intrinsic resistance, acquired resistance, genetic mutation, and horizontal gene transfer [31]. Intrinsic resistance describes when a bacterium survives an antibiotic by changing its structural or functional components. Acquired resistance involves bacteria gaining the ability to resist a particular antibiotic through a new genetic mutation or horizontal gene transfer. Genetic mutations that alter cellular and metabolic pathways render the bacterium unrecognizable to the antimicrobial agent. Horizontal gene transfer through transformation, transduction, or conjugation confers the spread of AMR genes between bacteria [31].

In livestock, common mechanisms of AMR involve enzymatic degradation of antibiotics, modification of antibiotic targets, horizontal gene transfer, and efflux pumps [32]. Enzymatic degradation of antibiotics, a common mechanism in Gram-negative bacteria, involves breaking down or inactivating antibiotics before they exert their effect, rendering them inactive. An example is beta-lactamase production by *Escherichia coli* and *Salmonella* strains in livestock that hydrolyze beta-lactam antibiotics such as penicillin and cephalosporins [33]. Modification of antibiotic targets entails bacteria altering the molecular target of the antibiotic, which prevents the drug from binding and exerting its effect. Alteration in bacterial target proteins induces changes to ribosomes, DNA gyrase, or other proteins that are targeted by antibiotics. For example, mutations in the ribosomal target can lead to resistance to macrolide antibiotics [34]. In pigs and cattle, some *Staphylococcus aureus* strains have acquired the *mecA* gene that modifies penicillin-binding protein (PBP) and leads to the production of alternative penicillin-binding protein 2a (PBP2a). This protein has a reduced affinity for beta-lactam antibiotics, including methicillin, compared to normal PBP. This reduces the efficacy and effectiveness of methicillin and other beta-lactam antibiotics against the infected bacteria [35]. This results in antibiotic resistance. Strains of *S. aureus* that have acquired the *mecA* gene are classified as methicillin-resistant *S. aureus* (MRSA).

Bacteria can alter their cell membrane permeability to prevent entry of the antibiotics. This may be due to changes in the structure of the cell membrane or the expression of efflux pumps, which actively pump antibiotics out of the cell. This mechanism is commonly used by Gram-negative bacteria, where their outer membrane is particularly adept at using efflux pumps to expel antibiotics. Efflux pumps actively pump antibiotics out of the bacterial cell, reducing their concentration within the cell. Some bacteria can prevent antibiotics from entering the cell in the first place (limited uptake) [34]. Limited uptake of antibiotics is due to the structure and functions of the LPS layer in gram-negative bacteria, which provides a barrier to certain types of molecules and gives innate resistance to certain groups of large antimicrobial agents [36]. For example, hydrophobic drugs such as rifampicin and fluoroquinolones can enter easily, but not the hydrophilic drugs in *mycobacteria* have a high lipid content. Contrarily, *Mycoplasma* and related species are intrinsically resistant to all drugs that target the cell wall [37]. Reygaert WC has comprehensively summarized these mechanisms with specific examples of AMR [34]. Some bacteria may develop a slow-growing phenotype (small colony variant), making them less susceptible to antibiotics that act on rapidly dividing cells [38]. This occurs due to mutations in a part of the genome affecting the electron transport and thymidine biosynthesis, leading to decreased ATP synthesis. The slow growth of bacteria renders antibiotics less effective, and aids in developing AMR, and recurrent infections [38].

Horizontal gene transfer confers antibiotic resistance through conjugation, transformation, and transduction [39]. Conjugation is the direct transfer of genetic material between bacteria. Transformation involves a bacterium acquiring extracellular DNA, and transduction occurs when a bacteriophage transfers DNA to the bacterium. In pigs and poultry, *Escherichia coli* has utilized horizontal gene transfer with the *mcr-1* gene to develop resistance to colistin, a last resort antibiotic [35]. Bacteria can also use efflux pumps to remove drugs before they reach lethal concentrations within the cell. *Salmonella* and *Campylobacter* are common pathogens in poultry that use the CmeABC efflux pump to expel the fluoroquinolones class of antibiotics, leading to prolonged infection [16].

Mutations in gyrase and topoisomerase IV are the most common cause of quinolone resistance [40]. A recent study in Nigeria analyzed human and animal stool samples that tested positive for Campylobacter and found higher resistance profiles of the livestock isolates. The resistance was greatest for beta-lactams (42%), followed by FQs (41%), tetracyclines (15%), and macrolides (2%) [41]. Another recent study in China sampled swine feces and wastewater from 21 swine farms in seven provinces of China and found that all isolates showed FQ resistance: resistance to norfloxacin (43%), ciprofloxacin (47.6%), ofloxacin (47%), and levofloxacin (38.8%) [42].

The primary cause of macrolide bacterial resistance is post-transcriptional methylation of bacterial 23S ribosomal RNA, typically gained through horizontal gene transfer [43]. In a Canadian study, samples from commercial feedlot animals affected with bovine respiratory disease were collected, and 90.2% of the isolates of *M. haemolytica*, *M. bovis*, *P. multocida*, *T. pyogenes*, and *H. somni* were found to be resistant to macrolides [44]. There are two well-characterized mechanisms of tetracycline resistance, including alteration in ribosomal protection proteins or efflux pumps [45]. One study compared the fecal flora of conventionally raised feedlot steers and those raised without antimicrobials. The results suggested a significantly higher prevalence of tetracycline resistance genes in samples of the conventionally raised group [46].

Antibiotic-resistant pathogens tend to gather in high numbers, known as reservoirs. Reservoirs of resistant bacteria can be found both inside livestock bodies as well as in the environment. In livestock, reservoirs are often found in the gut microbiome of livestock due to the favorable growing conditions and the plethora of microorganisms present in the region [47]. Environmental reservoirs are found in various locations; however, some of the primary areas are wastewater, aquaculture habitats, and agricultural drainage. This can have profound effects and accumulation of bacteria in seafood that is consumed by humans and other animals. Another primary location of environmental reservoirs is in animal manure, which can lead to further dispersal of these antibiotic-resistant bacteria to the wild animal population [47].

Furthermore, looking at regional disparities of antibiotic resistance in livestock, studies have shown that the highest antibiotic usage quantities are seen in Asia and South America [48]. It is also estimated that by 2040, the highest rises in antibiotic usage will be in Asia and Africa, with minimal rises in Europe and North America due to the stricter regulations in these regions [48]. Most of the antibiotic usage comes in poultry and pig farming. Another factor contributing to the rise in antimicrobial resistance is intensive farming. Studies have shown that intensive farming systems tend to show higher levels of antimicrobial resistance as well as a greater diversity of the overall resistance [49]. This is particularly relevant because intensive farming systems are becoming increasingly popular in developing countries as their populations continue to rise while the amount of available land for farming continues to decrease [49]. The results from these studies show major implications for the steady rise in antibiotic resistance worldwide.

The accumulation of these antibiotics inside the animals can have dramatic effects. Chronic exposure to antibiotics can cause weakened immune systems and increased rates of chronic infection in animals. In a study involving prolonged chlortetracycline use in calves, the gut microbial balance was affected by the loss of beneficial butyrate-producing bacteria like *Lachnospiraceae* [50]. Another study involving long-term antibiotic exposure (ampicillin, metronidazole, neomycin, vancomycin) in mice demonstrated the disruption of gut microbiota, leading to impaired immune function, cell-mediated responses, and associated cytokines, rendering the mice susceptible to *Candida albicans* infection [50]. A rise in chronic infection rate in animals leads to increased animal mortality, which can be worsened due to the gene transfer between animals. This can also diminish the quality and quantity of the antigens that can be used from livestock in the production and development of vaccines [51]. Another often-overlooked method of spreading includes the transmission to domestic companion animals. Animals such as dogs and cats can have reservoirs that begin either with contact with affected animals or the humans they interact with. This further exacerbates the spread of pathogens and can lead to infections and disease in companion animals [52]. Overall, mortality rates of animals increase with continuous exposure and administration of antibiotics.

## 5. Impact of Antibiotic Resistance on Humans

Before diving deeper into the pathogenic bacteria commonly transmitted to humans, it is essential to understand the variety of ways through which humans can be exposed to these bacteria, conferring antibiotic resistance. The transfer of antimicrobial-resistant pathogens can occur in different ways. One way is through the consumption of contaminated animal-based products, especially milk, meat, and eggs. The livestock feed used can commonly include antibiotic-resistant bacteria, which can be transferred to humans through the consumption of the products coming from that livestock [53]. Antibiotic-resistant bacteria can also have favorable breeding environments due to some farms having crowded and unsanitary living conditions for the animals. This can have a large impact on the production of milk products, as the bacteria can be present in machinery used in processing. They can also contaminate food products through air and water pollution [53]. Additionally, these bacteria can be easily transmitted from the animals to the workers on these farms because of their consistent daily exposure to the animals and environment. These pathogens are then spread to other humans through horizontal gene transfer, which contributes to the rapid nature of the transmission [54]. AMR in humans poses a massive threat to public health, as the diminishing efficacy of antimicrobials allows the development of infections that are difficult to treat, leading to prolonged illnesses, higher healthcare costs, and increased mortality rates.

Looking at the specific resistant bacteria transmitted, the most common pathogenic bacteria that is found in animal products is *Escherichia coli*. Typically, *E. coli* is found within the environment animals are living in, ranging from direct methods such as the soil in the region, which can come in contact with livestock feed through indirect methods, including water run-off and heavy metal presence in the habitat [55]. *E. coli* has been found to be transferred through the consumption of poultry, cattle, and pigs as well as their derived products. These resistant genes are then largely spread from human to human via horizontal gene transfer. Studies have shown that many strains of *E. coli* found in livestock are heavily resistant to streptomycin (up to 70%), a common antibiotic used to treat bacterial infections. However, other strains that are resistant to various other treatment options include ampicillin, amoxicillin/clavulanate, cefotaxime, ticarcillin, ciprofloxacin, trimethoprim, and tetracycline [56].

Another common pathogen conferring resistance in humans is *Campylobacter*, leading to gastroenteritis in humans. *Campylobacter* is generally cultivated in environments that are exposed to environmental pollutants, have large amounts of animal carcasses and excretions, and involve water transmission. It is commonly found in poultry, sheep, cattle, and swine, which can also be given to other animals through the food chain, further exacerbating the spread of the bacteria [57]. *Campylobacters* can lead to antibiotic resistance because of their ability to exchange genetic material with each other and other bacteria. This pathogen has very prominent resistance and virulence in humans and has various survival mechanisms to avoid the human immune response [58].

Overall, when looking at the effect of these bacteria on human health, it is important to note the wide variety of bacterial pathogens that can lead to detrimental effects in humans. This effect may be due to the bioaccumulation of pathogens in humans. Chronic exposure and accumulation can lead to severe allergic reactions as well as direct toxicity [59]. Allergies can manifest due to chronic exposure to these pathogens and are typically present with skin rashes, vasculitis, and, at times, hemolytic anemia. Allergic reactions often come from the consumption of contaminated milk and meat, especially pork [60]. Direct toxicity manifests in many forms, including hepatotoxicity, fertility issues, destruction of normal flora, and carcinogenicity. Hepatotoxicity can be due to antibiotics such as penicillin, oxacillin, cloxacillin, and flucloxacillin via cholestasis, whereas ceftriaxone induces hepatotoxicity through cholestasis and gallstones [61]. Flora, especially in the stomach, can be destroyed with exposure to ionophores that lead to gastrointestinal issues and disturbances. This can also create immune system deficiencies as well as renal issues [62]. Fertility issues can occur due to the increased number of mutations that arise with antibiotic resistance [50]. This mutagenicity leads to major implications on public health, as it can affect the current population as well as future offspring, leading to a rapid spread and rise of antibiotic resistance [60]. Lastly, some residues from sulfamethazine, oxytetracycline, and furazolidone can have carcinogenic effects secondary to drug resistance. The carcinogenic effects are expected to rise to 10 million deaths by 2050 [60,63].

## 6. Determining AMR Resistance and Choosing Antibiotics

Phenotypic methods, PCR-based methods, isothermal amplification methods, and DNA microarrays are conventional methods for AMR diagnosis. Genome sequencing and metagenomics, including pyrosequencing, whole genome sequencing, a combination of short and long read whole genome sequencing, nanopore sequencing, mass spectrometry (MALDI-TOF), and Fourier transform infrared (FTIR) spectroscopy, are non-conventional methods for AMR diagnosis. More advanced microfluidic technologies for AMR diagnostics include spectroscopy-based, colorimetric-based, pH-based, multiplexing, and single-cell sequencing. Non-conventional and microfluidic techniques are time-saving technologies. The newer technologies are beneficial in overcoming the limitations of conventional techniques, including low sensitivity, need for sample pretreatment steps, lack of automation and portability, and incapacity of microorganism detection [64]. The commonly used method for determining antibiotic resistance is culturing the sample in the presence of antibiotics and looking for the sensitivity and resistance of specific pathogens to a wide range of antimicrobial agents (antimicrobial susceptibility test) [65]. This test will tell us about the antibiotics that can be used against microbial agents. Following this, it is important to determine the dose of the antibiotic to be used. Minimum Inhibitory Concentration (MIC) is the lowest concentration of an antibiotic required to inhibit the growth of a specific microorganism most effectively. Culture and MIC tests indicate the sensitivity and resistance to an antibiotic for a particular organism [66].

## 7. Approaches to Attenuate Antibiotic Use in Livestock

A multifaceted approach combining prevention, responsible use, and alternatives is needed to reduce the use of antibiotics in livestock. Improving biosecurity, hygiene, and vaccination, along with exploring natural supplements, vaccines, and new antimicrobial technologies, are various strategies to attenuate antibiotic use in livestock. Improving biosecurity involves keeping herds away from potential sources of infection, restricting access to farms, and controlling vectors like rodents and flies. Consistent cleaning and disinfection of barns, effective waste management, and optimal ventilation are crucial for preventing pathogen spread, thereby decreasing antibiotic use. Vaccinating animals against specific diseases can reduce the need for antibiotics. Judicious use of antimicrobials and regular monitoring of antibiotic resistance patterns can help guide antibiotic choices and identify emerging resistance threats. The use of antibiotics in livestock may also be attenuated by using natural supplements like vitamin D, multivitamins, minerals, alternative growth promoters, probiotics, and natural chemicals found in plants, like safflowers and peppers, which help in improving animal health and the immune system, altogether decreasing antibiotics use. Educating the public and farmers about the dangers of antibiotic resistance and the importance of responsible antibiotic use, along with policies for restricting antibiotic use, promoting responsible use, and incentivizing the development of alternatives, are crucial in attenuating the use of antibiotics [67,68,69].

Selective Dry Cow Therapy (SDCT) is a strategy that reduces antibiotic use by only treating dairy cows with mastitis at dry-off, rather than administering antibiotics to all cows. This approach, unlike Blanket Dry Cow Therapy (BDCT), which treats all cows and targets those at higher risk of infection, leading to a more efficient and cost-effective use of antimicrobials. SDCT programs use various methods to identify cows at high risk of intramammary infection (IMI) at dry-off, such as individual somatic cell count (SCC) records, mastitis history, and milk culturing [70,71,72]. A recent study reported a significant reduction of 22% on average in overall antimicrobial use for udder health when cows were selectively allocated for antimicrobial treatment [73]. Huey et al. [70] conducted a study including 19 farmers to investigate the barriers and facilitators for SDCT. The study found increasing incidence of mastitis, lack of infrastructure, peer pressure, and a perceived lack of preventive advice as barriers to SDCT, while regulatory pressure, targeted veterinary consults, and high standards of farm hygiene as facilitators for SDCT. This suggests that educating farmers on AMR and preventive strategies is needed. Educating farmers is important because SDCT significantly reduces the overall amount of antibiotics used on dairy farms, leading to a decrease in the selective pressure for resistant bacteria. 

A study by Lipkens et al. [73] evaluated whether or not implementing SDCT, compared to BDCT, on commercial dairy farms reduces antimicrobial consumption without negatively affecting future performance. The study reported a significantly lower antimicrobial use for udder health between drying off and 100 days in milk in SDCT compared to BDCT. These findings suggest the role of SDCT in reducing antibiotics in livestock without jeopardizing udder health and milk yield. Another study [74] evaluated the effect of dry cow therapy on the AMR profile of mastitis pathogens post-calving with 382 cows. The study reported an increased resistance percentage from dry-off to post-calving in coagulase-negative *Staphylococcus* species isolates from antimicrobial drug-treated cows. The highest percentage increase of 12.2% was recorded for penicillin. The study also observed an increased resistance against cephalothin, oxacillin, and ceftiofur in coagulase-negative *Staphylococcus* species isolates at both dry-off and post-calving in cows receiving dry cow therapy from the same drug class, or a class with a shared resistance mechanism. However, resistance to tetracycline was associated with any antimicrobial drug therapy at dry off. 

Further, resistance to Penicillin decreased in cows receiving any antimicrobial drug (AMD) therapy at dry-off compared to those not receiving it. Another cross-sectional study [75] reported a reduced use of antimicrobials around the dry-off between 31% and 66% with SDCT. It was also observed that multiparous cows are at higher risk of mastitis compared to primiparous cows. Risk was also associated with the season of dry-off, and cows drying off in the spring, summer, and fall had lower odds compared to winter months in the USA. Another study [76] checking the efficacy of SDCT compared to BDCT on bovine udder in healthy animals confirms the possibility of selective drying without new intramammary infection risk or somatic cell count at calving. These findings suggest the role of SDCT as an effective approach to minimizing antibiotic use while maintaining animal health and welfare. These findings suggest SDCT as an effective approach to reduce the use of antimicrobials and AMR, but studies with larger cohorts are needed to further confirm the effectiveness of SDCT for its wider use at community levels.

Herbal therapy offers potential alternatives to reduce reliance on antibiotics in livestock by leveraging the natural antimicrobial and immunostimulating properties of plants. Herbs like red clover, thyme, oregano, and sage have been shown to possess antimicrobial activity, while others like garlic, Echinacea, and nettle can enhance immune function [77,78]. These herbs either have antimicrobial properties, increase the bioavailability of antibiotics, or reduce the time of stay in the milk, altogether minimizing the development of AMR. For example, fibrosin increases the bioavailability of ceftizoxime in milk and helps eliminate it faster in goats. This may reduce the duration of antibiotic exposure, minimizing the chance of resistance. The herbs used in herbal therapy can work synergistically with antibiotics, increasing their effectiveness against certain bacterial species. Herbal therapy can work as probiotics and symbiotics, improving gut health and reducing disease incidence and the use of antibiotics [79,80,81,82]. For example, Sar et al. [79] reported that the use of a single oral dose of the commercial mammary protective polyherbal drug causes non-persistence of ceftriaxone and reduces the persistence of ceftizoxime in the milk of goat, cows, and buffaloes. A study [83] used garlic powder and probiotics in calves and reported improved immune response, lower fecal scores, and fewer days of diarrhea compared to control groups. Another study [84] using chestnut powder in newborn calves reported improved diarrhea in treated calves compared to controls, with significantly higher incidences of diarrhea. Wageningen Academy (https://edepot.wur.nl/453731; assessed on 24 May 2025) reported that the use of a product containing formic acid, citric acid, and herbal extracts (chamomile, plantain, thyme, and sundew) could reduce infection levels in pigs, leading to a decrease in antibiotic use. This strategy increased the life of piglets and the prevalence of turning them into adults, which previously were dying due to lung infections.

These studies suggest the beneficial role of SDCT and herbal therapy; however, further studies are warranted to investigate the most effective dose, timing, frequency, and combinations using larger trials with larger cohorts. While low doses of herbal extracts may be safer for preventing infections, higher concentrations may be needed for curative effects, which could potentially lead to unintended consequences [78]. Thus, more research is needed to fully understand the potential of herbal therapies in reducing antimicrobial resistance and to develop effective and safe products for use in livestock.

## 8. Current Gaps and Recommended Guidelines

Limited understanding of the relative contributions of different sources (livestock waste, human waste, etc.) of antibiotics and resistant bacteria into the environment, the role of the environment in the evolution of resistance, and the impact of resistant bacteria on human and animal health are key research gaps in the literature regarding antimicrobial resistance (AMR) in livestock. Understanding how these substances behave in the environment and how they contribute to the evolution of resistance is needed [85]. Additionally, there is a lack of comprehensive risk assessments that encompass all potential transmission pathways of resistant bacteria from livestock to humans, including through consumption of meat, direct contact, and environmental contamination [86]. More research is needed to understand the overall human and animal health impacts of exposure to resistant bacteria from the environment. The economic impact of AMR on livestock, including costs related to treatment, reduced productivity, and potential losses in the food chain, needs to be better understood [85,87]. Further research is also needed to evaluate the efficacy of different interventions, including technological, social, economic, and behavioral approaches, to mitigate the emergence and spread of AMR in the environment and livestock [85,88]. Improved data collection and surveillance systems are needed to track antibiotic use and resistance prevalence in livestock and to share this information with stakeholders [86]. In addition to this, following the recommended guidelines to decrease the use of antibiotics is also needed and should be implemented.

Using antibiotics only when necessary, under veterinary supervision, and for the recommended duration; avoiding using antibiotics for growth promotion or prevention of disease in healthy animals; and prioritizing antibiotics that are least important to human medicine are common recommendations to decrease the use of antibiotics in livestock as well as to decrease the emergence of AMR. A study reported that restricted antibiotic use in food-producing animals reduced antibiotic-resistant bacteria in these animals by up to 39% (commonly ranging between 10 and 15%) [89]. WHO guidelines on the use of medically important antimicrobials in food-producing animals recommend complete restriction of the use of antimicrobials for growth promotion and disease prevention in healthy livestock, as well as reductions in the use of medically important antimicrobials in food-producing livestock [90]. The guidelines also recommend that antimicrobials classified as critically important for human medicine should not be used for control of the dissemination of a clinically diagnosed infectious disease identified within a group of food-producing animals. The guidelines also recommend that antimicrobials classified as Highest Priority Critically Important for human medicine should not be used for the treatment of food-producing animals with a clinically diagnosed infectious disease [90].

To attenuate AMR, the U.S. Department of Agriculture (USDA) has provided National Guidelines (https://www.usda.gov/antimicrobial-resistance-overview-amr, assessed on 27 May 2025). National strategies for antimicrobial resistance (AMR) in livestock focus on reducing the emergence and spread of drug-resistant organisms through a multi-faceted approach, including education, surveillance, research, infection prevention and control, and optimizing antimicrobial use. These strategies are often framed within a “One Health” approach, recognizing the interconnectedness of human, animal, and environmental health. National Strategic Action Plan (NSAP) on AMR suggests that educating livestock producers, veterinarians, and the public about responsible antimicrobial use, infection prevention, and biosecurity practices is crucial to attenuating AMR. Establishing robust surveillance systems to track antimicrobial use, resistance patterns, and the emergence of new resistance mechanisms is essential for informing policy and interventions. Implementing strong biosecurity measures on farms and in veterinary settings to prevent the spread of resistant bacteria is key. Promoting the judicious use of antimicrobials in livestock, including avoiding unnecessary use, using the correct dose and duration, and ensuring proper disposal of unused medications, is critical. Effective national strategies require collaboration between government agencies, veterinary organizations, livestock producers, and other stakeholders (as per USDA and NSAP) [91].

The U.S. National Strategy for Combating Antibiotic-Resistant Bacteria, released in 2013, focuses on improving the tracking of resistant bacteria, extending the life of current antibiotics, and accelerating the development of new antibiotics and interventions [92]. The European Union’s “One Health” action plan includes measures to reduce antimicrobial use in livestock, harmonize monitoring of antimicrobial resistance, and promote good practices in animal health [91]. The Food and Agriculture Organization (FAO) has developed a framework for national strategies on AMR, emphasizing the importance of collaboration, strengthening surveillance, and promoting appropriate antimicrobial use in livestock production (FAOLEX Database LEX-FAOC202557, https://apps.who.int, assessed on 27 May 2025). The World Organisation for Animal Health (WOAH) provides guidelines and recommendations for national strategies on AMR, including monitoring antimicrobial use, harmonizing surveillance, and promoting responsible use of antimicrobials in veterinary medicine (https://www.woah.org/app/uploads/2021/03/en-amr-strategy-final.pdf, assessed on 27 May 2025). This suggests global cooperation and involvement to attenuate AMR in livestock.

## 9. Future Perspective

Although the therapeutic use of antibiotics is important to ensure the health of livestock animals and the safety of animal products for human consumption, their excessive and sub-therapeutic use for growth promotion is increasing the rate of emergence of antibiotic-resistant genes. Both scientific and governmental/regulatory strategies must be implemented to hinder the progression of antimicrobial resistance (AMR) within livestock and reservoirs and prevent its transmission to humans.

Recommendations have been developed by the intergovernmental organization Centre for Agriculture and Bioscience International (CABI) regarding how to address the issue of AMR: massive global awareness campaigns, improving hygiene to prevent infection transmission, reducing unnecessary use of antimicrobials and their spread into the environment, promote new diagnostics to reduce unnecessary use of antibiotics, and developing new vaccines and new types of modified antibiotics [93]. In the United States, the FDA and the USDA are responsible for regulating antibiotic residues and ensuring they remain below unsafe levels designated by the agencies. In addition to removing FDA approval of antimicrobial use for growth enhancement and feed efficiency improvement, the FDA has changed the marketing status of antimicrobials to ensure veterinary oversight and consultation via Veterinary Feed Directives and prescriptions. 

Antimicrobial peptides (AMPs) are next-generation antibiotics that are biodegradable, offering an approach to reducing AMR. Most organisms, from humans to bacteria, endogenously produce AMPs as pathogenic defense mechanisms [94]. One advantage to AMPs is their broad spectrum of antimicrobial activity against bacteria, fungi, parasites, and viruses [95]. Another advantage is their non-specific mechanisms of action on bacterial membranes, which do not induce AMR [96]. One challenge that may be presented with the biodegradability of AMPs is their stability within organismal environments, as seen in cases regarding bovine mastitis [94]. However, they remain a promising alternative due to the slower rate of bacterial adaptation to AMPs compared to conventional antibiotics. A recent study has reported the use of bovine lactoferrin in chickens as an antibiotic alternative to prevent Clostridium perfringens type A/G-induced avian necrotic enteritis in view of the restriction of the use of antibiotics in chickens [97].

Conventional antibiotics are typically broad-spectrum, which can lead to the indiscriminate killing of commensal bacteria that may be beneficial to an organism. Gene editing via CRISPR/Cas systems offers a way to program antimicrobials that selectively kill AMR bacteria based on their genetic sequences, sparing beneficial commensal bacteria. They can reduce the colonization of target populations in vivo when delivered via phage capsids. Additionally, AMR bacteria can be re-sensitized to antibiotics by curing plasmids carrying resistance genes [98]. However, the use of CRISPR/Cas systems may face resistance in its implementation due to differing public acceptance of in vivo gene editing.

Another approach to AMR is the development of DNA and mRNA vaccines. This strategy aims to prevent disease development and transmission among livestock populations, reducing the need for antibiotic treatment. Since it is the overuse of antibiotics that causes selective pressure for AMR, reducing the overall administration of antibiotics to animals is crucial. Vaccination of animals utilizes the insertion of a gene of interest into a plasmid, along with the necessary machinery for replication and protein synthesis. Insertion of the plasmid into the host causes antigenic protein production and a subsequent immune response. Currently, the majority of vaccines used in livestock are antiviral, although there have been several vaccines developed or under development to prevent infection from bacterial pathogens such as *M. hypopneumoniae* in swine and *Salmonella* and *Pasteurella* in poultry. The World Organization for Animal Health (OIE) report from 2019 recommended the prioritization of vaccine development for certain diseases to reduce antimicrobial use in livestock. In poultry, these diseases included those caused by *E. coli* (yolk sac infection, air sacculitis, cellulitis), *C. perfringens type A* (necrotic enteritis), as well as coccidiosis, infectious bronchitis virus, and infectious bursal disease virus. In swine, pathogens included *Streptococcus suis*, *Pasteurella multocida*, *Actinobacillus pleuropoeumoniae*, *Swine influenza virus*, *E. coli*, *Rotaviruses*, and *Haemophilus parasuis*, among others [23,67,99]. The recommendations outlined by the report offer guidance on which vaccine developments would have the largest impact on AMR development in poultry, swine, cattle, and fish.

DNA vaccines present some obstacles, such as in vivo efficacy and stability. Additionally, single administration of DNA vaccines is often insufficient to induce robust immune responses, requiring the need for boosters, as seen in poultry for avian pathogens [100]. Such disadvantages may be overcome by utilizing certain physical and biological carriers, such as a polycation-based delivery system [100]. mRNA vaccines offer specific advantages, as they may be non-infectious, non-integrating, and capable of natural degradation, rapid production, and B and T cell immune response induction [101].

Bacteriophages are viruses that can infect bacteria, and they have been used therapeutically due to their lytic and lysogenic cycles, which can be exploited to insert desired genes and lead to subsequent protein expression. A recent study shows promising results in the use of phages against *S. aureus*, the bacteria responsible for mastitis in cattle [102]. In swine, phage therapy saw a reduction in colonization of Salmonella by more than 99% in a group of neonatal pigs [103]. Phage therapy is appealing due to its high specificity, ability to multiply and evolve at infection sites, and its lower economic cost, and should garner more global attention as an approach to AMR [104]. Potential limitations to phage therapy include the narrow spectrum of available host strains, in vivo stability, immune responses, and phage resistance [23] (Table 1).

An antigen vaccine is a type of vaccine that contains a specific antigen, which is a molecule that triggers an immune response in the body. This antigen can be a part of the disease-causing organism, a weakened or inactive form of the organism, or even the blueprint for producing the antigen. Vaccines work by exposing the body to the antigen without causing the disease, allowing it to learn to recognize and fight off the real infection in the future. Antigen vaccines containing specific antigens can help livestock fight antimicrobial resistance by preventing infections and reducing the need for antibiotics. By stimulating the immune system to recognize and neutralize pathogens, vaccines can prevent bacterial infections and thus decrease the pressure on antibiotic use. This reduces the selection pressure for resistant strains, slowing the development and spread of antimicrobial resistance [105,106,107].

## 10. Conclusions

Antibiotic resistance in livestock is a major public health concern because the overuse of antibiotics in animals contributes to the emergence and spread of antibiotic-resistant bacteria, which can then be transferred to humans through various routes. This includes direct contact with animals, exposure to contaminated manure, consumption of undercooked meat or dairy products, and even through the environment. To decrease livestock antibiotic resistance, focus on reducing antibiotic use, improving livestock husbandry practices, and implementing alternative approaches to disease prevention and treatment. Key strategies to mitigate AMR include limiting antibiotic use to targeted treatments under veterinary guidance, promoting good hygiene and sanitation, and exploring vaccination and other preventative measures.

## Figures and Tables

**Table 1:** Advantages and limitations of various techniques used to mitigate antimicrobial resistance (AMR) in livestock.

Approach	Advantages	Limitations
Antimicrobial Peptides (AMPs)	Biodegradable Broad-spectrum activity Naturally produced by most organisms Non-specific mechanisms reduce AMR development Slower bacterial adaptation compared to antibiotics	Stability issues in vivo Biodegradability may limit therapeutic persistence
CRISPR-based Tools	Precision targeting of AMR genes Spares commensal bacteria Can re-sensitize bacteria by removing resistance plasmids Can be delivered via phage capsids	Requires advanced delivery systems Still in early stages of livestock application
Vaccines	Prevents infection and reduces antibiotic need DNA vaccines induce antigen-specific immunity mRNA vaccines are non-infectious, rapidly produced, and naturally degradable	DNA vaccines may lack in vivo efficacy Often require boosters Delivery systems needed to improve stability and immune response
Bacteriophages	Highly specific to target bacteria Can multiply at infection site Low economic cost	Requires careful strain matching Regulatory and scalability challenges remain Immune responses -phage resistance

## Data Availability

Not applicable.
